# Longitudinal associations between paternal mental health and child behavior and cognition in middle childhood

**DOI:** 10.3389/fpsyg.2023.1218384

**Published:** 2023-11-01

**Authors:** Sherri Lee Jones, Christina Caccese, Kelsey P. Davis, Jimin Lew, Guillaume Elgbeili, Catherine M. Herba, Julia Barnwell, Cindy Hénault Robert, Isabella Gavanski, Kristin Horsley, William D. Fraser, Deborah Da Costa, Jean R. Séguin, Tuong-Vi Nguyen, Tina C. Montreuil

**Affiliations:** ^1^Department of Psychiatry, Faculty of Medicine and Health Sciences, McGill University, Montreal, QC, Canada; ^2^Department of Psychiatry, Research Institute of the McGill University Health Center, McGill University, Montreal, QC, Canada; ^3^Department of Psychiatry, Douglas Research Center, Douglas Mental Health University Institute, McGill University, Montreal, QC, Canada; ^4^Integrated Program in Neuroscience, Department of Psychiatry, McGill University, Montreal, QC, Canada; ^5^Department of Psychology, Université du Québec à Montréal, Montreal, QC, Canada; ^6^Research Center, CHU Sainte-Justine Mother and Child University Hospital Center, Montreal, QC, Canada; ^7^Department of Pediatrics, Human Genetics and Pharmacology & Therapeutics, McGill University, Montreal, QC, Canada; ^8^Department of Psychology, McGill University, Montreal, QC, Canada; ^9^Department of Obstetrics and Gynecology, Centre de Recherche du CHU de Sherbrooke, University of Sherbrooke, Sherbrooke, QC, Canada; ^10^Department of Physical and Occupational Sciences, Faculty of Medicine and Health Sciences, McGill University, Montreal, QC, Canada; ^11^Department of Psychiatry and Addictology, Université de Montréal, Montreal, QC, Canada; ^12^Lady Davis Institute for Medical Research, Jewish General Hospital, Montreal, QC, Canada; ^13^Reproductive Psychiatry Program, Department of Psychiatry and Obstetrics and Gynecology, McGill University Health Centre, Montreal, QC, Canada; ^14^Department of Educational and Counselling Psychology, Faculty of Education, McGill University, Montreal, QC, Canada

**Keywords:** paternal depressive symptoms, paternal anxiety symptoms, child cognition, child behavior, child development

## Abstract

**Introduction:**

Paternal mental health has been associated with adverse consequences on offspring psychosocial development, and family environmental factors may partly explain those associations. To clarify this, we need comprehensive prospective studies, particularly in middle-childhood when the child enters school and is expected to make use of behavioral and cognitive skills as part of their interactions and learning.

**Method:**

Using data from a sub-sample of the prospective 3D birth cohort study comprised of mother-father-child triads, and a follow-up of the parents and the children at 6–8 years of age (*n* = 61; 36 boys, 25 girls), we examined whether paternal anxious and depressive symptoms measured during the pregnancy period (i.e., prenatally) or concurrently when the child was assessed at 6–8 years old were associated with children's cognition/behavior.

**Results:**

In contrast to our hypotheses, we found that greater prenatal paternal depressive symptoms predicted fewer child behavioral difficulties; and that greater concurrent childhood paternal depression or anxiety symptoms were associated with higher child full-scale IQ, controlling for the equivalent maternal mental health assessment and parental education. Father parenting perception did not mediate these associations, nor were they moderated by maternal mental health at the concurrent assessment, or paternal ratings of marital relationship quality.

**Discussion:**

These findings suggest that higher symptoms of paternal mental health symptoms are associated with fewer child behavioral difficulties and higher cognitive performance in middle childhood. Potential clinical implications and future research directions are discussed.

## 1. Introduction

Recent evidence suggests that both paternal and maternal mental health play a role in shaping the developmental trajectory of the child. Not everyone reacts to perceived stress or manages challenging situations in the same way. Stress is defined as a stimulus that is unpredictable or uncontrollable to the organism, eliciting a physiological, behavioral, and cognitive stress response (Koolhaas et al., 2011). Anxiety is defined as the pathological response to one's inability to manage this stress response adaptively (Duval et al., 2015). While it is well-documented that early life maternal depression, perceived stress, and anxiety are associated with adverse child outcomes (Barona et al., 2017; Lin et al., 2017; Cook et al., 2018; Tuovinen et al., 2018; Vehmeijer et al., 2018), the associations between paternal mental health and child developmental outcomes require further investigation (Wanless et al., 2008; Paulson et al., 2009; Sweeney and MacBeth, 2016; Gentile and Fusco, 2017; Cui et al., 2020; Challacombe et al., 2023). It is unclear, for example, which aspects of paternal mental health (i.e., symptom type, level of severity, or timing) are associated with child development, over and above maternal mental health. Moreover, most of the research on paternal mental health has focused on paternal depression, neglecting other important risk factors such as anxiety and perceived stress (Fisher, 2016).

A detailed characterization of paternal mental health factors in relation to the child's development may be particularly important during middle childhood. This critical developmental period, beginning at 6 years old, is characterized by an increase in the incidence of several psychiatric disorders among children (Kessler et al., 2007). This is perhaps not surprising given the important number of learning and adaptive demands that are placed on children at this age to acquire a host of novel cognitive, social, and behavioral skills during their transition from kindergarten to elementary school (Dworkin, 1989; Campbell, 2011). Indeed, a meta-analysis has reported that paternal mental health issues present during pregnancy, particularly depression, increased the risk of behavioral problems and doubled the risk of suffering from psychiatric disorders in school-aged children (Gentile and Fusco, 2017). Paternal depression was associated with lower prosocial skills, greater peer problems, and a higher incidence of psychiatric disorders in 6- to 7-year-olds (Ramchandani et al., 2008). Moreover, chronic exposure to parental mental health symptoms (from the prenatal period and persisting into middle childhood) may amplify associations with child outcomes over time (Rajyaguru et al., 2021). While prenatal paternal mental health symptoms and related conditions such as assisted reproductive techniques, poor nutrition, substance use, stress, and lack of exercise may also lead to alterations in sperm that, in turn, adversely affect children (Rodgers et al., 2013; Ibrahim and Hotaling, 2018; Yeshurun and Hannan, 2019; Hoek et al., 2020), longitudinal follow-up studies are lacking.

Associations between prenatal or childhood paternal mental health symptoms and child outcomes may interact with or be mediated by other environmental factors, particularly in the context of the transition to formal schooling. Maternal mental health status and the quality of the marital relationship may play a role in the child's development in terms of cognitive functioning, sense of self and agency, and emotional regulation (Fisher, 2016) and may interact with paternal mental health. Thus, while associations between paternal factors and child outcomes are hypothesized, one must also consider potential roles that environmental factors may play, such as interactions with maternal mental health, family conflict/discord, and differing parental styles or views (Flouri and Buchanan, 2003, 2004; Sarkadi et al., 2008; Wanless et al., 2008; Ferreira et al., 2016; Ragni et al., 2019). Indeed, higher self-reported measures of hostile/reactive parenting have been shown to mediate associations between maternal prenatal stress and higher child externalizing problems (Hentges et al., 2019). Thus, it is possible that paternal views of parenting may mediate any associations between paternal mental health symptoms and child cognitive/behavioral outcomes.

While there is evidence to suggest that paternal depression, perceived stress, and anxiety are associated with their offspring's development, knowledge gaps exist regarding the potential roles of factors such as (1) the type of paternal mental health symptom (i.e., anxiety, stress, and depression); (2) symptom timing (i.e., prenatally vs. in childhood); (3) symptom severity; and (4) other socio-environmental factors that may interact with paternal mental health or mediate the association between paternal mental health and the child's cognitive/behavioral outcomes.

As such, this study aimed to examine whether prenatal or concurrent paternal anxious and depressive symptoms are associated with the child's cognitive functioning and behavior at the ages of 6–8 years old, to assess the relative associations in terms of timing of mental health symptoms (i.e., prenatal vs. middle childhood), and to assess potential environmental factors that may moderate or mediate those relationships. We address several knowledge gaps by using prospectively collected data from mother–father–child triads, acquired from the first trimester of pregnancy up to childhood at 6–8 years of age. Assessments included anxious and depressive symptoms and perceived stress in both parents measured during pregnancy and in middle childhood, as well as standardized assessments of cognition and behavior in the child. Based on measures taken prenatally as well as when the child was in middle childhood, we hypothesized that (1) higher levels of paternal anxious/stress/depressive symptoms assessed prenatally and concurrently in childhood would predict lower cognitive scores, lower prosocial scores, and higher behavioral problems in the offspring during middle childhood while controlling for the equivalent maternal anxious/stress/depressive symptoms and parental education level; (2) associations between paternal concurrent mental health and offspring cognitive-behavioral outcomes in middle childhood would interact with those measured during the prenatal period; and (3) (a) maternal mental health and paternal ratings of marital relationship satisfaction would moderate and (b) paternal parenting perception would mediate the associations detected between paternal mental health and child development.

## 2. Methods

### 2.1. Participants

Participants were recruited from the existing 3D (Design, Develop, Discover) pregnancy cohort comprised of 2,366 mother–father–child triads, initiated by the Integrated Research Network in Perinatology of Quebec and Eastern Ontario (IRNPQEO; https://www.irnpqeo.ca/en/) (Fraser et al., 2016). In the context of the present study, perinatal assessments include parental mental health (first data collection of self-reported depression, anxiety, and stress symptoms, collected during the pregnancy) and psychosocial measures (reported during pregnancy: parental highest level of education; and at 24-months post-natally: Quality of Marriage Index, and parenting perceptions). We conducted a follow-up assessment during middle childhood, when children were between 6 and 8 years old, focusing on spontaneous pregnancies and mother–father–child trios from the 3D study who agreed to complete both the 3D-transition follow-up study when their child turned 6 years old (Rioux et al., 2021) and to participate in additional data collection for our affiliate paternal study. In total, 61 families (36 boys, 25 girls; 50 whites) completed all the procedures required by the paternal protocol. This sub-group did not differ significantly from the initial 3D cohort or the larger 3D-Transition study, in terms of parental age, education, or ethnicity.

The present study was approved by the Research Ethics Board (REB) at the McGill University Health Centre (MUHC) and the CHU Sainte-Justine Mother and Child University Hospital Center, and conformed to the Declaration of Helsinki's standards. Written informed consent was obtained from parents for their portion of the study and on behalf of their children. Verbal assent was obtained from the child participants.

### 2.2. Procedures

The prenatal paternal and maternal anxiety, stress, and depressive symptom measures, sociodemographic measures (i.e., education and household income, parental alcohol consumption, and nicotine use), parental parenting perceptions data, and quality of marriage index (measured at 24 months after the birth of the child) were acquired from the existing 3D cohort's dataset (Fraser et al., 2016).

Concurrent parental mental health data (Beck Depression Inventory, Beck Anxiety Inventory) and children's cognitive (Wechsler Intelligence Scale for Children) and behavioral assessments (Strengths and Difficulties Questionnaire) were obtained concurrently as part of a research visit when the child was between 6 and 8 years old. We also collected data on the parents' and child's full health history (e.g., health conditions, diagnoses, child pubertal examination, parental alcohol consumption, smoking, and drug use), as well as a physical examination to determine the child's overall health (i.e., vital signs, height, weight, and skin fold measurement). Children also provided saliva samples and structural brain images acquired with magnetic resonance imaging, which are not included in the context of the project goals reported here.

### 2.3. Instruments

#### 2.3.1. Parental mental health

As part of the 3D study (Fraser et al., 2016), mothers completed self-report questionnaires on their mental health during each trimester of pregnancy, whereas fathers only provided these measures once during the second-trimester visit. In the context of the current study, the first assessment data were used. Depressive symptoms were measured using the Center for Epidemiological Studies Depression Scale (CES-D). Maternal CES-D (10-item) was completed during the first trimester, and paternal (4-item) CES-D was collected during the second trimester. CES-D has been shown to have very high internal consistency, adequate test–retest reliability, and good construct validity when used in the general population (Radloff L. S., 1977; Miller et al., 2008). The four-item version of the CES-D has been shown to be highly correlated with longer versions and validated both in English and French (Melchior et al., 1993; Zauszniewski and Graham, 2009), in the current sample, Cronbach's α = 0.81 for mothers and α = 0.612 for fathers on the CES-D. These reliability measures are consistent with the literature. Lower reliability in fathers vs. mothers has been reported by others (e.g., Schudlich and Cummings, 2007), and reliability decreases with fewer items (Carpenter et al., 1998).

Prenatal anxiety was measured only during the second trimester for both mothers and fathers, using a screening questionnaire developed by Séguin and Freeston for anxiety disorder symptoms based on DSM-IV criteria (Anxiety Screening Inventory—STR; Séguin et al., 2000; Shapiro et al., 2017). Cronbach's α in the current sample was 0.783 for maternal STR and 0.851 for paternal STR.

The four-item self-reported perceived stress scale (PSS) was measured in mothers during the first trimester and in fathers during the second trimester. The PSS has been shown to have good internal consistency and validity (Cohen, 1994; Lee, 2012). Cronbach's α in the current sample was 0.858 for maternal PSS and 0.713 for paternal PSS.

As part of the paternal follow-up study when their children were 6–8 years old, parents also completed self-report questionnaires measuring depressive symptoms using the Beck Depression Inventory (BDI) (Beck et al., 1961) and anxiety symptoms using the Beck Anxiety Inventory (BAI) (Beck et al., 1988). The BDI has been extensively validated across both clinical and community samples with high internal consistency (Cronbach's α = 0.86 for psychiatric patients; Cronbach's α = 0.81 for non-psychiatric participants) and high concurrent validity with clinical ratings (*r* = 0.72 for psychiatric patients; *r* = 0.60 for non-psychiatric participants) (Beck and Steer, 1984; Beck et al., 1988). Within the current sample, Cronbach's α = 0.917 for mothers and α = 0.853 for fathers on the BDI. The BAI has been shown to have excellent internal consistency (Cronbach's α = 0.94) and good test–retest reliability (*r* = 0.67) (Fydrich et al., 1992). Within the current study, Cronbach's α = 0.879 for mothers and α = 0.791 for fathers on the BAI.

#### 2.3.2. Child cognition and behavior

Seven subscales of the Wechsler Intelligence Scale for Children—Fifth Edition (WISC-V; Block Design, Similarities, Matrix Reasoning, Digit Span, Coding, Vocabulary, and Figure Weights) were administered by a trained child neuropsychologist to the child participants (ages 6–8 years old) to assess cognition. The full-scale IQ was derived from these subscales. A verbal comprehension index score was also derived from the similarities and vocabulary subscales, and a fluid reasoning index score was derived from the matrix reasoning and Figure Weights subscales. The WISC-V has been found to provide a reliable, valid (in both English and French), and fair estimation of intelligence and cognitive constructs in children 6–16 years old (Pearson, 2017).

To measure the behaviors of the child participants, we asked the parent attending the research visit to complete the Strengths and Difficulties Questionnaire during middle childhood (SDQ version for 4–10 years of age) (Goodman, 1997, 2001) while the child was undergoing cognitive testing. The SDQ has been shown to have good internal consistency, test–retest reliability (Cronbach's α = 0.73) (Goodman, 2001), inter-rater agreement (parent and teacher), construct validity, concurrent validity, and predictive validity, with the total difficulties scale, in particular, having high sensitivity and specificity for identifying child psychopathology (Goodman et al., 2010; Stone et al., 2010; Mieloo et al., 2012). The SDQ comprises five subscales: emotional problems, peer problems, conduct problems, hyperactivity/inattention symptoms, and prosocial skills (reverse scored). The sum of all subscales (excluding the prosocial skills subscale) provides a total difficulties score. In the current sample, Cronbach's α for total difficulties was 0.829, and for prosocial was 0.691. The emotional and peer problems subscales are summed to form an internalizing score (Cronbach's α in the current study = 0.658), and the conduct problems and hyperactivity/inattention subscales are summed to form an externalizing score (Goodman et al., 2010) (Cronbach's α in the current study = 0.856).

#### 2.3.3. Parenting perceptions

Each parent self-reported the Parental Cognitions and Conduct Toward the Infant Scale (PACOTIS) at three timepoints: 3, 12, and 24 months after birth. Because no parenting measure was collected during the middle childhood assessment, the latest assessment (i.e., 24 months) was used in the context of the current study. PACOTIS assesses self-reported parenting perceptions and behaviors of parents toward their infants and includes assessments of parental self-efficacy, parental overprotection, perceived parental impact, parental hostile/reactive behaviors, and parental warmth/affection on a 10-point scale (Boivin et al., 2005). In this study, we reverse-coded the hostile/reactive scale and created a total score by summing all subscales, where higher scores represent more positive self-reported parenting perceptions. Cronbach's α was calculated on the current sample for the maternal PACOTIS (α = 0.759) and for the paternal PACOTIS (α = 0.105). The paternal subscales with poor reliability were impact (α = −0.007) and overprotection (α = 0.022), whereas the remaining scales had good reliability: hostile/reactive α = 0.799, warmth/affection α = 0.732, and self-efficacy α = 0.833.

#### 2.3.4. Quality of Marriage Index

When the child was 3, 12, and 24 months old, each parent also completed the Quality Marriage Index (QMI), a six item self-report questionnaire where participants rated the level of support received from their partner across different domains (e.g., child care, household chores; Norton, 1983). Marital relationship quality assessed by the QMI has been shown to be both theoretically and empirically relevant to studies of child development (e.g., by predicting attachment security; Fincham, 1998). In the current study, we used available data from the 24-month assessment because no measure of relationship quality was assessed at the 6- to 8-year-old assessment. Cronbach's α in the current sample for the maternal QMI was α = 0.823, and for the paternal, QMI was α = 0.713.

### 2.4. Statistical analyses

Analyses specific to aims 1, 2, and 3 are described in more detail in Sections 2.4.1, 2.4.2, and 2.4.3, respectively.

All the families in this sample completed parental mental health, as well as child cognitive and behavioral measures; thus, a no-missing data protocol was applied. Both parental and child measures were evaluated for outliers (>2.5D from the mean), and no cases were found (i.e., all cases were included).

Descriptive analyses were first conducted to assess the distribution of the variables of interest, and Pearson's correlations were run to assess the simple associations between all predictors and outcomes.

Covariates in all analyses were selected based on the existing literature. The equivalent maternal mental health symptoms and highest parental education level (maternal and paternal combined) were included as covariates in all analyses. Based on the existing literature, other potential covariates that were considered included the sex of the child, gestational age at birth, the child's age in days, parental prenatal care, and concurrent alcohol and nicotine use. These latter variables were not associated with child outcomes of interest (raw scores on the SDQ or WISC-V) and therefore were not included as covariates in subsequent statistical models.

To adjust for multiple comparisons when analyzing subscales of SDQ, a correction using false discovery rate (FDR; Benjamini–Hochberg method; Benjamini and Hochberg, 1995) was applied to analyses, where “*q*” was set at a standard level of 0.05 and where the threshold of significance (*Q*) is then equal to (*i*/*m*)^*^q (*I* = rank in terms of significance, *m* = total number of tests). All regression-based mediation and moderation analyses were conducted using PROCESS version 2 (http://processmacro.org/). Significance levels were set at a *p*-value of ≤0.05. All data were analyzed using IBM SPSS Statistics 24.

#### 2.4.1. Aim 1: paternal mental health—Prenatally and in childhood—And child development

We conducted multiple linear regressions for each paternal predictor variable (CES-D, PSS, STR, BDI, and BAI) and child outcome (SDQ and WISC-V). For initial analyses, we examined only the total difficulties and prosocial skills subscale scores extracted from the SDQ, as well as the full-scale IQ and two sub-indices from Verbal Comprehension and Fluid Reasoning (WISC-V). Each combination of child outcome and paternal predictor was run in a separate regression. If the analysis on an outcome's total scale was significant, *post-hoc* analyses were conducted on each of its subscales to determine which one(s) drove the effect. Two sets of analyses were run: the associations between prenatal paternal mental health and child outcomes, and the associations between concurrent paternal mental health and child outcomes. The maternal mental health measure and parental education at the same time points as paternal mental health were entered as covariates.

#### 2.4.2. Aim 2: timing of paternal depression and anxiety

For those paternal–child relationships found to be significant in Aim 1, we assessed whether the timing of paternal depression and anxiety (i.e., measured during pregnancy or concurrently in childhood) was associated with child outcomes by using multiple regression to test whether prenatal paternal symptoms moderated the relationship between current paternal depression and anxiety and child outcomes, and vice versa. In other words, the statistical models that were significant in Aim 1 were rerun with the addition of a prenatal x concurrent paternal mental health interaction term.

#### 2.4.3. Aim 3: the role of other environmental factors

To further examine the associations between paternal mental health symptoms detected in Aim 1 and child development detected in Aim 1, we examined potential moderation or mediation by other environmental factors. Equivalent maternal mental health symptoms (i.e., maternal score on the same measure at the same time point) and paternal ratings of marital satisfaction measured when the child was 24 months were entered as moderators in separate models, as their roles are less clear, and standardized assessments of these variables are scarce. Additionally, father–child perceived parenting self-reported at 24 months was considered a mediator based on the previous literature (e.g., Hentges et al., 2019). The maternal equivalent was controlled for in these models.

## 3. Results

### 3.1. Sample characteristics

Descriptive statistics for selected characteristics, including predictors and outcomes, are included in Table 1, and Pearson's correlations for selected determinants and outcomes can be found in Table 2. All parental mental health measures (CES-D, PSS, STR, BDI, and BAI) and child outcomes (SDQ and WISC-V) were in the normal range, with paternal self-reported scores tending to be lower than maternal scores, and most levels of parental education reported were at the university level. As expected, greater maternal mental health symptoms (depression and anxiety) were associated with adverse child behavioral outcomes, both prenatally (CES-D: *n* = 55, *r* = 0.298, *p* = 0.027; STR: *n* = 52, *r* = 0.298, *p* = 0.032) and current (BDI: *n* = 61, *r* = 0.477, *p* < 0.0001; BAI: *n* = 61, *r* = 0.360, *p* = 0.004), but not with cognitive outcomes; the parental highest level of education was correlated with the child full-scale IQ (*n* = 61, *r* = 0.456, *p* < 0.0001) and fluid reasoning (*n* = 61, *r* = 0.347, *p* = 0.006).

**Table 1:** Participant characteristics.

	***N***	**Mean**	**SD**	**Range**
**Child**				
Chronological age (months)	61	79.98	6.49	66.83–100.89
Gestational age (days)	60	272.85	12.35	223–293
WISC full scale	61	112	12.87	77–140
WISC verbal comprehension	61	75.03	25.09	14–99.6
WISC fluid reasoning	61	68.37	23.16	12–99.5
SDQ total difficulties	61	8.1	5.64	0–28
SDQ prosocial	61	8.62	1.64	3–10
**Maternal prenatal assessment**				
CES-D	55	7.85	5.02	0–21
PSS	56	4.16	3.04	0–12
STR	52	6.62	5.13	0–19
**Maternal concurrently assessed at child's 6–8 years of age**				
BDI	61	7.2	7.84	0–36
BAI	61	5.97	6.67	0–34
**Paternal prenatal assessment**				
Paternal CES-D	58	0.83	1.3	0–6
Paternal PSS	58	3.48	2.72	0–12
Paternal STR	58	5.84	5.65	0–28
**Paternal concurrently assessed at child's 6–8 years of age**				
Paternal BDI	55	3.91	4.47	0–17.3
Paternal BAI	55	3.86	4.27	0–14

WISC, Weschler Intelligence Scale for Children; SDQ, Strengths and Difficulties Questionnaire; CES-D, Center for Epidemiological Studies Depression Scale; PSS, Perceived Stress Scale; BDI, Beck Depression Inventory; BAI, Beck Anxiety Inventory.

Note that Paternal CES-D was measured with the 4-item CES-D scale, whereas Maternal CES-D was measured with the 10-item CES-D scale.

**Table 2:** Pearson's correlations between main predictors and outcomes (*n* = 61).

		**SDc_ebdtot**	**SDc_prosoc**	**WISCc_Full**	**D_CESD_T**	**D_PSS_T**	**D_STR_T**	**M_CESD_T **	**M_PSS_T**	**M_STR_T**	**BDI_D_T**	**BAI_D_T**	**BAI_M_T**	**BDI_M_T**	**QMI_M_T8**	**QMI_D_T8**	**REM_T_T8**	**REP_T_T8**	**HiEduTotal**
SDc_prosoc	Pearson's *r*	−0.478^***^	–																
	*p*-value	9.852e-5	–																
WISCc_Full	Pearson's *r*	−0.240	0.147	–															
	*p*-value	0.062	0.258	–															
D_CESD_T	Pearson's *r*	−0.222	0.205	−0.072	–														
	*p*-value	0.094	0.123	0.591	–														
D_PSS_T	Pearson's *r*	−0.044	0.111	−0.118	0.670^***^	–													
	*p*-value	0.743	0.406	0.379	8.754e−9	–													
D_STR_T	Pearson's *r*	−0.023	0.079	0.013	0.663^***^	0.661^**^	–												
	*p*-value	0.864	0.553	0.922	1.473e−8	1.650e−8	–												
M_CESD_T	Pearson's *r*	0.298^*^	−0.182	−0.037	0.204	0.261	0.202	–											
	*p*-value	0.027	0.184	0.786	0.138	0.057	0.142	–											
M_PSS_T	Pearson's *r*	0.118	−0.098	0.091	0.243	0.190	0.195	0.724^***^	–										
	*p*-value	0.387	0.471	0.506	0.074	0.164	0.153	4.213e−10	–										
M_STR_T	Pearson's *r*	0.298^*^	−0.116	−0.070	0.072	0.124	0.263	0.512^***^	0.314^*^	–									
	*p*-value	0.032	0.413	0.624	0.614	0.387	0.062	2.010e−4	0.028	–									
BDI_D_T	Pearson's *r*	−0.027	0.144	0.244	0.483^***^	0.362^**^	0.484^***^	−0.106	−0.108	0.046	–								
	*p*-value	0.844	0.293	0.072	2.848e−4	0.008	2.775e−4	0.465	0.451	0.760	–								
BAI_D_T	Pearson's *r*	0.114	−0.151	0.203	0.250	0.314^*^	0.515^***^	0.096	−0.017	0.328^*^	0.489^***^	–							
	*p*-value	0.409	0.272	0.136	0.074	0.023	9.308e−5	0.507	0.907	0.026	1.515e−4	–							
BAI_M_T	Pearson's *r*	0.360^**^	−0.097	0.009	0.013	0.133	0.162	0.094	0.089	0.265	0.312^*^	0.503^***^	–						
	*p*-value	0.004	0.457	0.943	0.925	0.319	0.225	0.496	0.513	0.058	0.020	9.221e−5	–						
BDI_M_T	Pearson's *r*	0.477^***^	−0.297^*^	0.049	−0.045	0.077	0.074	0.478^***^	0.440^***^	0.420^***^	0.105	0.432^***^	0.598^***^	–					
	*p*-value	1.030e−4	0.020	0.710	0.738	0.565	0.581	2.268e−4	6.802e−4	0.002	0.445	9.792e−4	3.637e−7	–					
QMI_M_T8	Pearson's *r*	−0.190	0.155	0.047	−0.427	−0.408	−0.513^*^	−0.113	−0.266	−0.301	−0.446	−0.428	0.071	−0.249	–				
	*p*-value	0.408	0.503	0.839	0.060	0.074	0.021	0.678	0.302	0.240	0.073	0.086	0.760	0.277	–				
QMI_D_T8	Pearson's *r*	−0.033	0.134	0.170	−0.645^**^	−0.473	−0.671^**^	−0.237	−0.347	0.095	−0.450	−0.028	0.070	0.111	0.686	–			
	*p*-value	0.900	0.608	0.515	0.007	0.064	0.004	0.394	0.205	0.747	0.080	0.919	0.790	0.671	0.089	–			
REM_T_T8	Pearson's *r*	−0.299^*^	0.237	−0.019	0.228	0.180	0.159	−0.498^***^	−0.491^***^	−0.079	0.299	0.109	0.037	−0.333^*^	−0.191	−0.046	–		
	*p*-value	0.046	0.117	0.903	0.141	0.248	0.307	7.868e−4	9.556e−4	0.624	0.061	0.504	0.811	0.025	0.512	0.886	–		
REP_T_T8	Pearson's *r*	0.153	−0.067	−0.168	−0.327	−0.154	−0.188	0.067	−0.127	0.080	−0.387^*^	−0.019	0.023	−2.600e−4	0.467	0.532	−0.192	–	
	*p*-value	0.341	0.679	0.293	0.042	0.350	0.251	0.689	0.446	0.652	0.016	0.909	0.889	0.999	0.079	0.092	0.292	–	
HiEduTotal	Pearson's *r*	−0.141	−0.054	0.456^***^	−0.206	−0.220	9.929e−4	−0.005	0.157	0.018	−0.002	−0.081	−0.045	0.094	0.167	0.196	−0.264	0.047	–
	*p*-value	0.278	0.677	2.204e−4	0.120	0.097	0.994	0.971	0.246	0.902	0.988	0.559	0.732	0.471	0.469	0.452	0.079	0.769	–

* p < 0.05.

** p < 0.01.

*** p < 0.001.

All measures were collected at the same time, upon or shortly after school entry, for children ~6–7 years of age. SDc_ebdtot, Strengths and Difficulties Questionnaire (SDQ) Total Difficulties score; SDc_prosoc, SDQ Prosocial score; WISCc_Full, Child total IQ; D_CES-D_T, Paternal Center for Epidemiological Studies Depression Scale (CES-D)-Total Score (T); D_PSS_T, Paternal Perceived Stress Scale (PSS)-Total Score; D_STR_T, Paternal anxiety scale- Total Score; M_CES-D_T, Maternal CES-D- Total Score; M_PSS_T, Maternal PSS- Total Score; M_STR_T, Maternal anxiety scale- Total Score; BDI_D_T, Paternal Beck Depression Inventory (BDI)- Total Score; BAI_D_T, Paternal Beck Anxiety Inventory (BAI)- Total Score; BAI_M_T, Maternal BAI- Total Score; BDI_M_T, Maternal BDI- Total Score; QMI_M_T8, Maternal Quality of Marriage Index (QMI) at 24 months postpartum; QMI_D_T8, Paternal QMI at 24-month postpartum; REM_T_T8, Maternal Parental Cognitions and Conduct Toward the Infant Scale (PACOTIS)-Total Score-at 24 months postpartum; REP_T_T8, Paternal PACOTIS-Total Score-at 24 months postpartum; HiEduTotal, Combined parental highest level of education.

Lower paternal ratings of the couple's relationship satisfaction measured at 24 months after the birth of the child (QMI) were correlated with higher paternal prenatal anxiety and depression symptoms. No correlations were detected between paternal QMI and current paternal anxiety and depression symptoms, nor with child behavioral symptoms at 6–8 years of age.

### 3.2. Aim 1: paternal mental health and child development

#### 3.2.1. Prenatal paternal mental health

**Figure 1:**
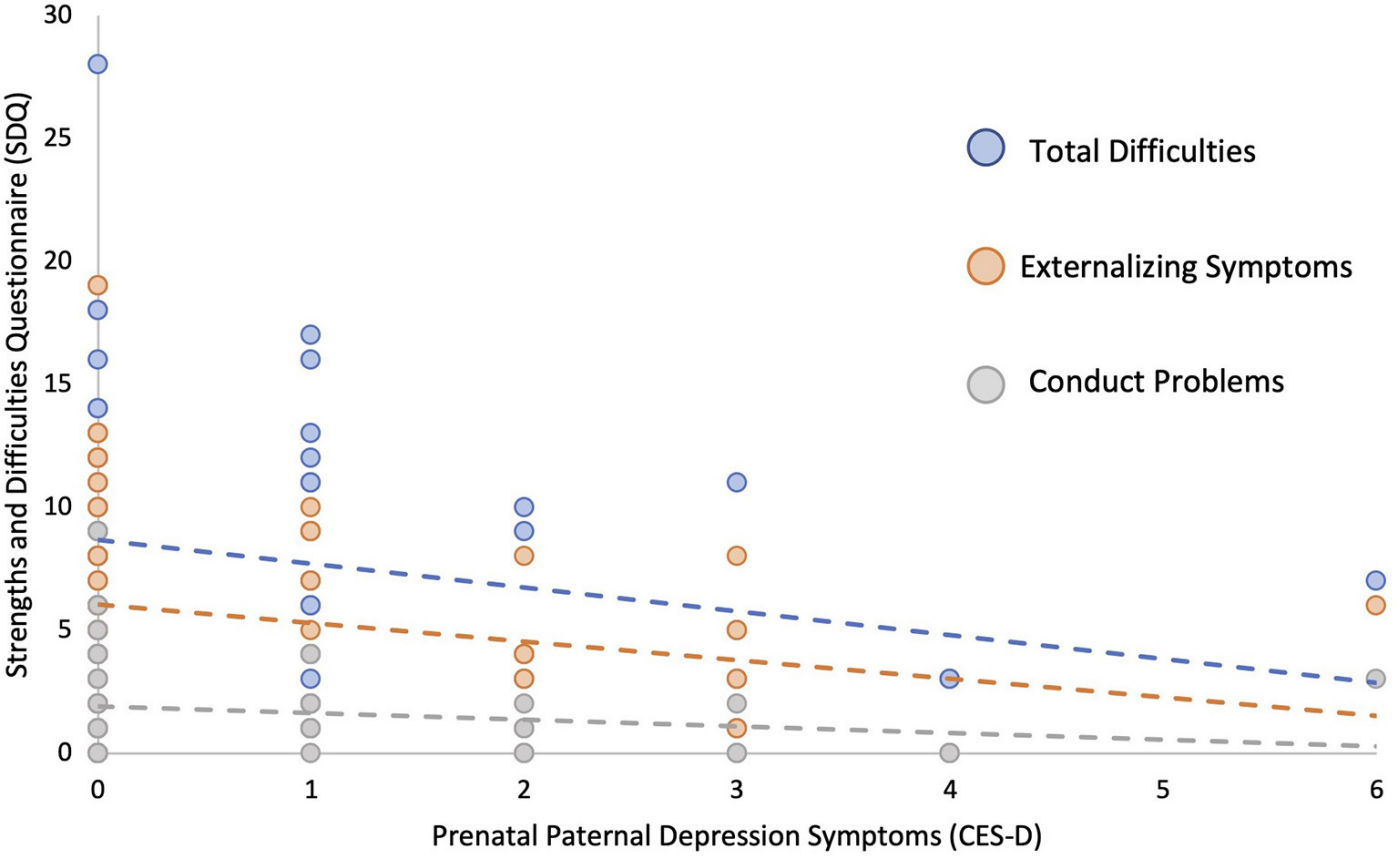
Higher prenatal paternal depression symptoms as measured by Center for Epidemiological Studies Depression Scale (CES-D) predicts lower child total difficulties, externalizing symptoms, and conduct problems as measured by the Strengths and Difficulties Questionnaire (SDQ).

Using linear regression models adjusted for prenatal maternal depression symptoms and parental highest level of education, higher prenatal paternal depressive symptoms (CES-D) were significantly associated with overall fewer behavioral/emotional difficulties in their children (β = −0.313, *p* = 0.023). *Post-hoc* analyses revealed that this relationship was primarily associated with externalizing (β = −0.312, *p* = 0.027, *q* = 0.054), as opposed to internalizing symptoms (β = −0.182, *p* = 0.191, *q* = 0.191), with higher paternal depressive symptoms predicting lower levels of externalizing symptoms such as conduct problems (β = −0.285, *p* = 0.041, *q* = 0.056) and hyperactivity/inattention (β = −0.275, *p* = 0.056, *q* = 0.056) in the child (see Figure 1 and Table 3). In contrast, no significant relationships were found between prenatal paternal levels of stress (PSS) or anxiety (STR) and child behavior or cognition when controlling for maternal anxiety symptoms and parental education level (*p* > 0.207, see Tables 4, 5).

**Table 3:** Regression analyses with prenatal paternal depression symptoms as a predictor of child cognitive and behavioral outcomes.

**Model**	***B***	**95% CI**	**β**	***t***	***p*-value**
**SDQ total difficulties**					
Paternal CES-D	−1.359	−2.521, −0.198	−0.313	−2.350	0.023^*^
Maternal CES-D	0.411	0.115, 0.706	0.362	2.789	0.007^*^
Parental education	−0.467	−1.058, 0.125	−0.206	−1.585	0.119
**SDQ prosocial**					
Paternal CES-D	0.321	−0.047, 0.689	0.247	1.752	0.086
Maternal CES-D	−0.079	−0.172, 0.015	−0.232	−1.688	0.098
Parental education	−0.027	−0.214, 0.160	−0.040	−1.688	0.098
**WISC-V full scale IQ**					
Paternal CES-D	0.921	−1.626, 3.469	0.094	0.726	0.471
Maternal CES-D	−0.136	−0.785, 0.512	−0.053	−0.423	0.674
Parental education	2.522	1.226, 3.819	0.497	3.907	< 0.001^*^

B, unstandardized beta; β, standardized beta. All analyses controlled for prenatal maternal depression symptoms and parental highest level of education. CES-D, Center for Epidemiological Studies Depression; SDQ, Strengths and Difficulties Questionnaire; WISC-V, Weschler's Intelligence Scale for Children Fifth Edition.

* p < 0.05.

**Table 4:** Regression analyses summarizing null findings of prenatal paternal perceived stress as a predictor of child cognitive and behavioral outcomes.

**Outcome**	***B***	**95% CI**	**β**	***t***	***p*-value**
SDQ total difficulties	−0.268	−0.860, 0.324	−0.130	−0.907	0.368
SDQ prosocial	0.075	−0.104, 0.253	0.122	0.837	0.406
WISC-V full-scale IQ	−1.56	−1.360, 1.047	−0.034	−0.261	0.795

All analyses controlled for prenatal maternal perceived stress and parental highest level of education. B, unstandardized beta; β, standardized beta.

**Table 5:** Regression analyses summarizing null findings of prenatal paternal anxiety as a predictor of child cognitive and behavioral outcomes.

**Outcome**	***B***	**95% CI**	**β**	***t***	***p*-value**
SDQ total difficulties	−0.172	−0.442, 0.098	−0.178	−1.280	0.207
SDQ prosocial	0.051	−0.032, 0.137	0.183	1.245	0.219
WISC-V full-scale IQ	0.114	−0.447, 0.676	0.052	0.410	0.684

All analyses controlled for prenatal maternal anxiety and parental highest level of education.

B, unstandardized beta; β, standardized beta.

#### 3.2.2. Childhood paternal mental health at 6- to 8-year-old assessment

**Figure 2:**
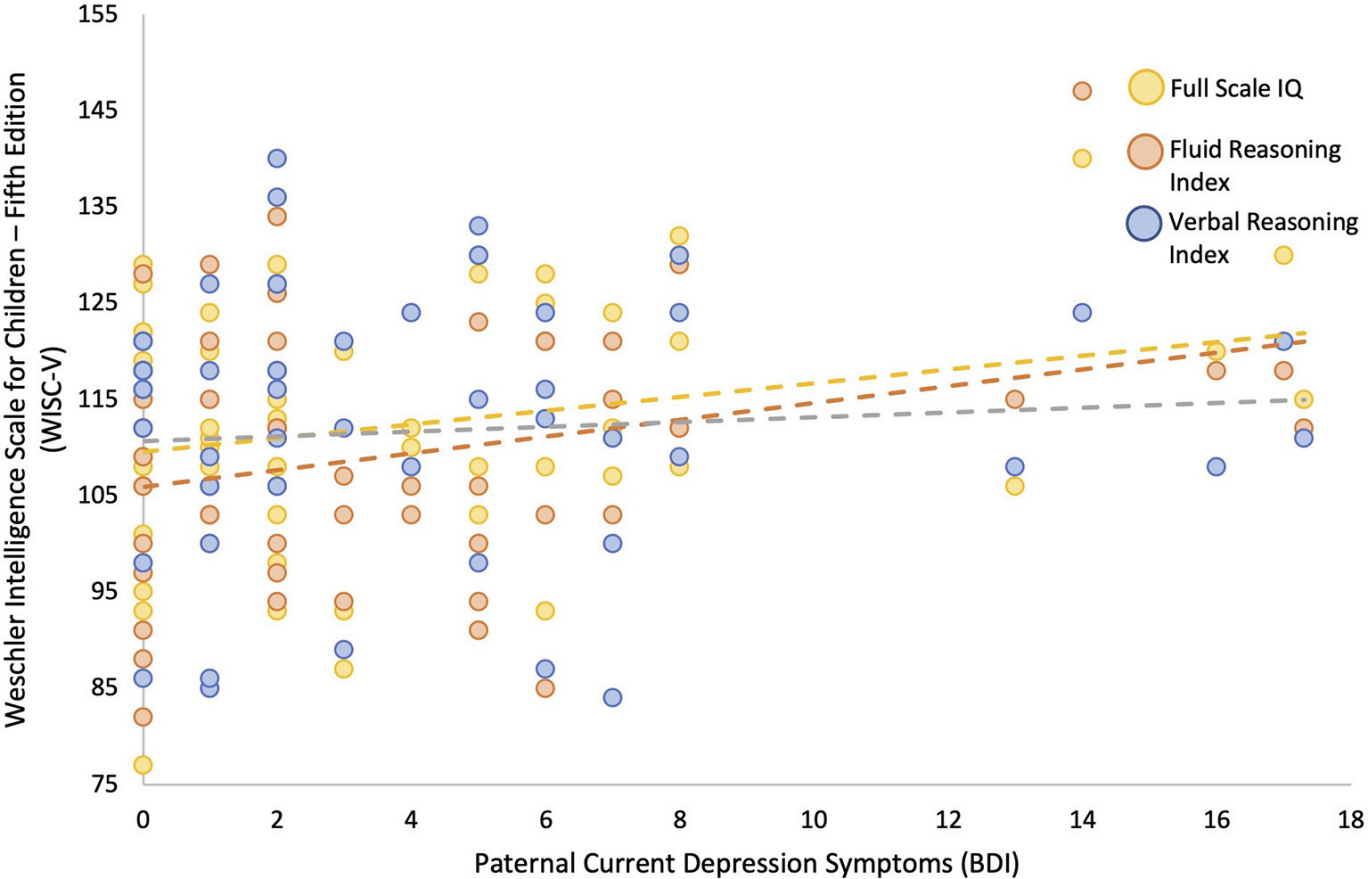
Higher concurrent paternal depression as measured by Beck Depression Inventory (BDI) at the 6–8 year-old child assessment predicts higher child full scale IQ and fluid reasoning performance as measured by Weschler Intelligence Scale for Children—Fifth Edition (WISC-V), but not verbal comprehension.

**Figure 3:**
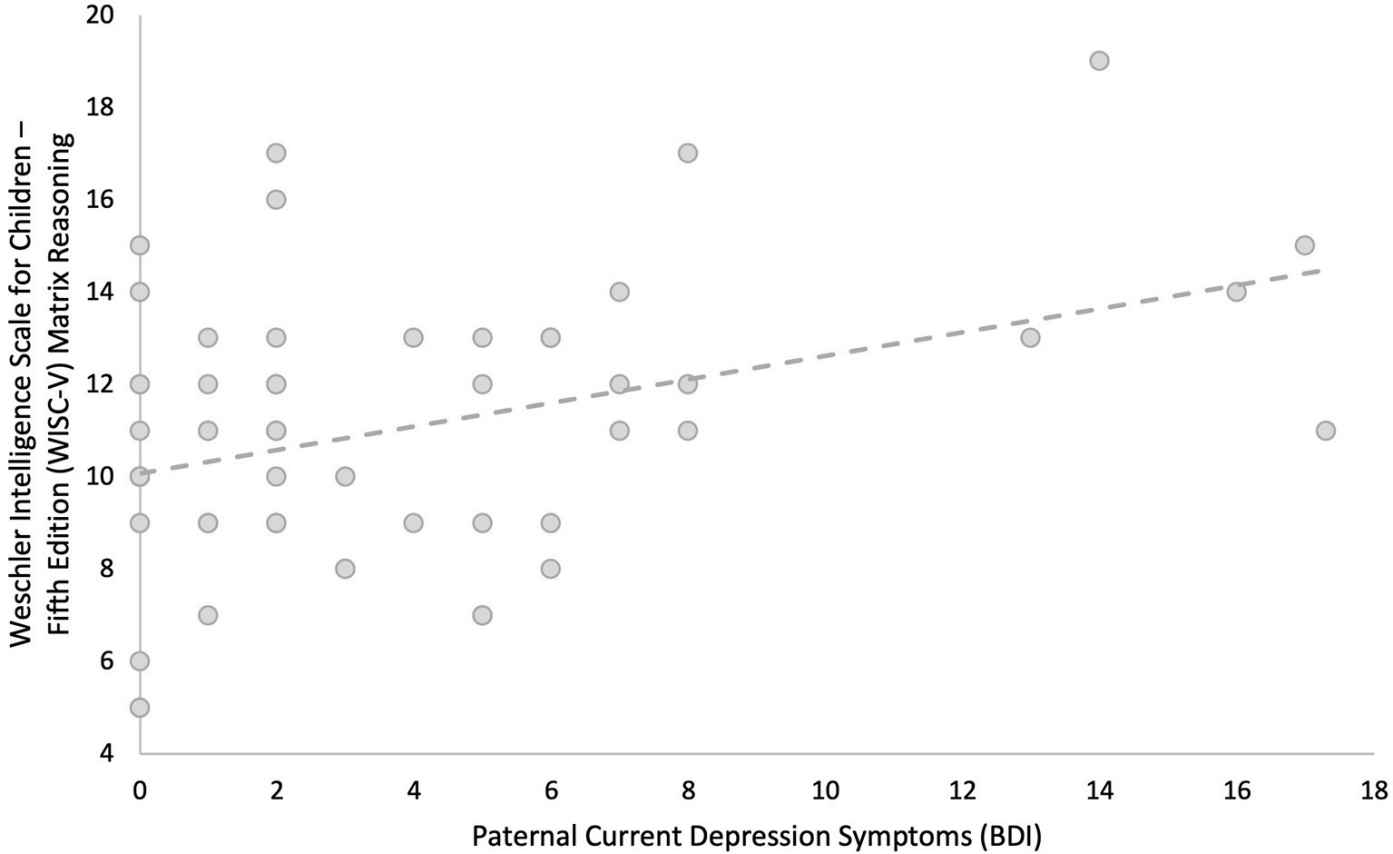
Higher concurrent paternal depression as measured by Beck Depression Inventory (BDI) at the 6–8 year-old child assessment predicts higher child performance on the matrix reasoning subscale of the fluid reasoning index as measured by Weschler Intelligence Scale for Children—Fifth Edition (WISC-V).

**Figure 4:**
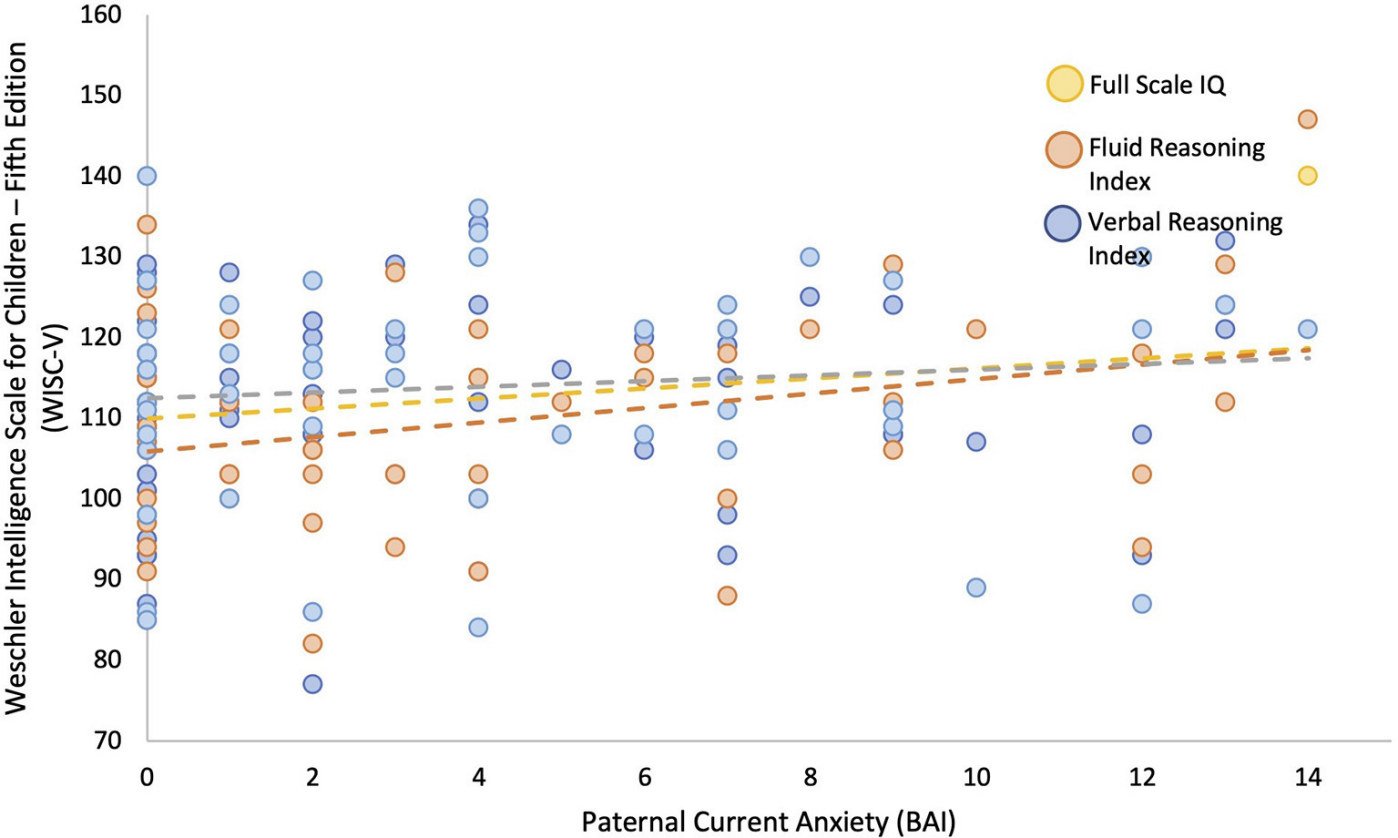
Paternal Anxiety Symptoms are Associated with Child Full Scale IQ and specifically the Fluid Reasoning Index (WISC-V). Higher current paternal anxiety as measured by Beck Anxiety Inventory (BAI) predicts higher scores on the child's full-scale IQ and higher fluid reasoning performance as measured by Weschler Intelligence Scale for Children—Fifth Edition (WISC-V).

**Figure 5:**
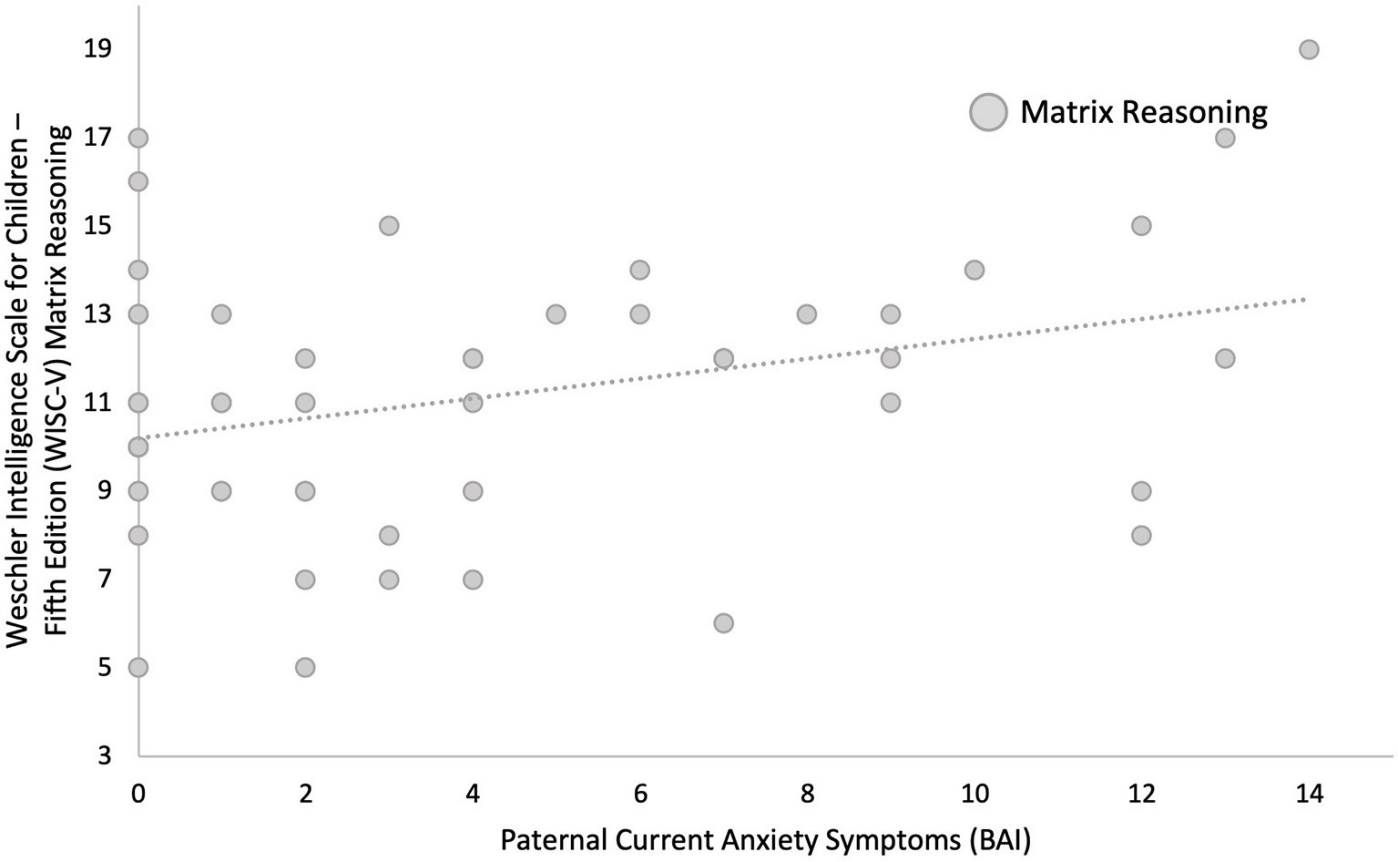
Higher concurrent paternal anxiety as measured by Beck Anxiety Inventory (BAI) during the child's 6–8-year old assessment predicts higher scores by the child on the matrix reasoning subscale of the fluid reasoning index as measured by Weschler Intelligence Scale for Children—Fifth Edition (WISC-V) at age 6–8.

Linear regression models adjusted for current maternal depressive symptoms and parental highest level of education showed that greater paternal depressive symptoms measured concurrently (BDI) during childhood were associated with higher child IQ (β = 0.246, *p* = 0.050). *Post-hoc* comparisons showed that this was primarily due to better performance on fluid reasoning (β = 0.294, *p* = 0.022, *q* = 0.044), which is composed of both the matrix reasoning (β = 0.377, *p* = 0.003, *q* = 0.007) and Figure Weights subscales (β = 0.066, *p* = 0.623, *q* = 0.623), as opposed to verbal comprehension (β = 0.039, *p* = 0.774, *q* = 0.774), composed of both the similarities and vocabulary subscales (see Figures 2, 3, and Table 6). Greater current paternal anxiety symptoms (BAI), after adjusting for current maternal anxiety symptoms and parental highest level of education, were also associated with higher full-scale IQ in the child (β = 0.304, *p* = 0.035), partly due to the associations with performance on the fluid reasoning index (β = 0.363, *p* = 0.014, *q* = 0.028) and its sub-component, the matrix reasoning subscale (β = 0.373, *p* = 0.013, *q* = 0.026; see Figures 4, 5, and Table 7). No other relationship using current paternal depression, stress, or anxiety symptoms to predict children's behavior or cognitive performance was significant (see Tables 6, 7).

**Table 6:** Regression analyses predicting child behavioral and cognitive outcomes from concurrent paternal depression symptoms.

**Model**	***B***	**95% CI**	**β**	***t***	***p*-value**
**SDQ total difficulties**					
Paternal BDI	−0.107	−0.421, 0.208	−0.081	−0.679	0.500
Maternal BDI	0.369	0.196, 0.542	0.513	−1.254	< 0.001^*^
Parental education	−0.377	−0.981, 0.227	−0.149	−1.254	0.216
**SDQ prosocial**					
Paternal BDI	0.067	−0.033, 0.167	0.178	1.348	0.184
Maternal BDI	−0.065	−0.120, −0.011	−0.316	−2.400	0.020^*^
Parental education	−0.040	−0.231, 0.151	−0.055	−0.419	0.677
**WISC-V full-scale IQ**					
Paternal BDI	0.719	0.000, 1.439	0.246	2.007	0.050^*^
Maternal BDI	−0.018	−0.413, 0.377	−0.011	−0.090	0.929
Parental education	2.396	1.016, 3.776	0.426	3.485	0.001^*^

All analyses controlled for current maternal depression symptoms and the parental highest level of education. BDI, Beck Depression Inventory; B, unstandardized beta; β, standardized beta.

* Significance at a p-value of ≤ 0.05.

**Table 7:** Regression analyses predicting child behavioral and cognitive outcomes from concurrent paternal anxiety symptoms.

**Outcome**	***B***	**95% CI**	**β**	***t***	***p*-value**
**SDQ total difficulties**					
Paternal BAI	−0.135	−0.547, 0.277	−0.099	−0.659	0.513
Maternal BAI	0.349	0.090, 0.608	0.404	2.704	0.009^*^
Parental education	−0.289	−0.945, 0.368	−0.114	−0.882	0.382
**SDQ prosocial**					
Paternal BAI	−0.048	−0.175, 0.078	−0.122	−0.765	0.448
Maternal BAI	−0.017	−0.097, 0.062	−0.070	−0.439	0.662
Parental education	−0.059	−0.261, 0.143	−0.081	−0.589	0.559
**WISC-V full-scale IQ**					
Paternal BAI	0.931	0.068, 1.794	0.304	2.165	0.035^*^
Maternal BAI	−0.250	−0.793, 0.293	−0.130	−0.925	0.360
Parental education	2.487	1.112, 3.863	0.442	3.630	0.001^*^

All analyses controlled for current maternal anxiety symptoms and parental highest level of education. BAI, Beck Anxiety Inventory.

* Significance at a p-value of ≤ 0.05.

### 3.3. Aim 2: paternal mental health symptom timing

We found no significant moderation of paternal mental health during pregnancy and childhood periods on the child's cognition or behavior. The timing of symptoms did not significantly moderate the relationship between paternal symptoms of depression and anxiety and child outcomes.

### 3.4. Aim 3: potential roles of environmental factors

#### 3.4.1. Maternal mental health as a moderator

Higher levels of childhood maternal anxiety tended to moderate the relationship between higher levels of childhood paternal depression and cognitive outcomes (*R*^2^ = 0.2974, *p* = 0.0613); however, this analysis failed to meet the statistical cutoff. No other relationship was significant or borderline significant.

#### 3.4.2. Paternal rating of Quality of Marriage assessed at 24 months as a moderator

After controlling for maternal ratings of the quality of the marriage and parental highest level of education, paternal ratings of marriage quality (QMI) did not moderate the observed relationships between paternal mental health symptoms and child behavioral problems or cognitive performance.

#### 3.4.3. Paternal parenting perception assessed at 24 months as a mediator

After controlling for maternal self-reported parenting perception (i.e., PACOTIS) and parental highest level of education, paternal self-reported parenting perception (PACOTIS) did not mediate any of the observed relationships between paternal mental health symptoms (depression/stress/anxiety) and child behavioral problems or cognitive performance.

## 4. Discussion

This study examined whether paternal mental health symptoms, reported during the prenatal period and concurrently at the follow-up assessment when the child was 6–8 years of age, were associated with child cognition and behavior. We also examined whether those associations were moderated by the timing of mental health symptoms and whether they could be explained by maternal mental health symptoms or other environmental factors. We found that higher paternal reports of prenatal depressive symptoms were associated with fewer behavioral/emotional difficulties in middle childhood at 6–8 years, controlling for maternal prenatal depressive symptoms and parental education. At the childhood assessment of 6–8 years of age, higher concurrent childhood paternal depressive and anxiety symptoms were both associated with higher cognitive performance in the child, controlling for the equivalent maternal mental health symptoms and parental education. Some potential environmental factors were considered, but we did not find support for any interactions between paternal mental health and maternal mental health paternal ratings of relationship satisfaction or any mediation by paternal perceived parenting. The results suggest that paternal mental health might not always have a negative association with child behavioral and cognitive outcomes, and our findings suggest that there is no strong evidence to support the moderating or mediating role of certain environmental factors in these associations.

Prenatal paternal depressive symptoms predicted fewer behavioral difficulties in the offspring at 6–8 years of age, over and above the contribution of maternal depressive symptoms to child behavioral difficulties. *Post-hoc* analyses revealed that these associations were primarily due to fewer externalizing symptoms, such as conduct and hyperactivity/inattention problems in the child. Importantly, we did not detect any interactions between maternal and paternal prenatal depressive symptoms and child behavioral difficulties. Though there have been reports of associations between paternal mental health and poorer child developmental outcomes, our data suggest that while controlling for maternal mental health, paternal mental health symptoms present pre- and/or post-natally will not necessarily result in negative developmental outcomes and psychopathology (Schetter and Tanner, 2012; Declercq et al., 2022) for all children, thus highlighting the relevance of individual variability. The basis for such individual differences is poorly understood, with emerging evidence pointing to aspects of caregiving in early childhood, including stable, responsive, and predictable caregiving (Belsky, 1984), thus resulting from the dynamic interaction between caregivers, their child, and other known social determinants.

Concurrent paternal symptoms of anxiety and depression measured at the child's 6–8 years of age assessment were both associated with higher full-scale IQ scores measured on the WISC-V, controlling for maternal concurrent anxiety or depressive symptoms (in the respective models) and parental education. These associations were primarily related to the child's performance on the fluid reasoning and matrix reasoning subscales. Fluid reasoning abilities are marked by the capacity to think logically and solve complex problems in novel situations independently of previously acquired knowledge (Weiss et al., 2016). The relative strength of associations between childhood paternal anxiety and depressive symptoms and full-scale IQ was similar, as seen in the standardized beta weights (~0.2–0.3 for each), which were significantly and moderately (*r* = ~0.5) correlated with each other, which shows that they each have a unique association beyond their shared variance. It is interesting to note that no concurrent association was detected between paternal depressive symptoms and childhood externalizing symptoms, in contrast to the associations with prenatal predictors.

The timing of paternal mental health symptoms was associated with different developmental outcomes in the child. The strength of the associations between the paternal mental health measures during the prenatal and middle childhood periods, although they differed in their respective associations with child outcomes, was similar in magnitude, as determined by the standardized betas (~0.2–0.3, equivalent to mild-moderate effect sizes). These data suggest that paternal prenatal mental health may be moderately predictive of fewer behavioral difficulties in middle childhood, whereas concurrent paternal mental health symptoms may predict higher cognitive performance. It is not clear when the switch of paternal associations with behavioral and cognitive performance occurs or what the mechanisms may be. We tested for potential interactions between prenatal and childhood paternal mental health measures, and no interactions were detected, suggesting distinct associations depending on the timing (prenatal vs. concurrent) of paternal mental health exposure and child developmental outcomes. Different conceptual models may account for those pre-. vs. post-natal relationships, though confirming the validity of such models was beyond the scope of the current study. For example, risk and resilience transmission from father-to-child may occur directly, along biological pathways (i.e., genetic and epigenetic sperm alterations related to paternal mental health pre-conception) (Bowers and Yehuda, 2016; Ibrahim and Hotaling, 2018; Yeshurun and Hannan, 2019; Hoek et al., 2020), or indirectly, through changes in antenatal maternal mental health (Ierardi et al., 2019). A prospective longitudinal birth cohort study with comprehensive pre-conception assessments on the father, including biopsychosocial measures and biospecimens of sperm as well as of the placenta after the birth of the child, would be needed to address factors involved in these probable time-sensitive risk transmissions. Such studies would help to determine more specifically, direct biological links between paternal, as well as maternal factors and child outcomes. Yet, in the absence of definitive findings on the timing of exposure to parental mental health issues to promote child developmental outcomes, there is a clear need to support the psychological wellbeing of parents both pre- and post-natally.

To address potential psychosocial mechanisms of these associations, we tested whether the equivalent maternal mental health or marital (relationship) satisfaction measures interacted with paternal mental health measures or whether paternal self-perceived parenting measures mediated the associations. None of these relationships were detected as statistically significant associations in our models. These findings in contrast to another study found that the associations between paternal post-natal (i.e., 8 weeks after the birth of the child) depression and more problematic child behaviors at 3.5 years old (also measured with the SDQ) were primarily mediated by family environmental factors that included maternal depressive symptoms and couple discord and dissatisfaction (Gutierrez-Galve et al., 2015). Some potential differences between these studies include the age of the child's behavioral measures and the proximity (timing) of environmental measures to the child's outcomes. We also note that the paternal parenting perceptions scale used (PACOTIS) had good internal item reliability for mothers, but poor internal item reliability for fathers overall. This is likely due to the poor reliability of the impact and overprotection subscales, suggesting that these subscales did not reliably capture the father's perceived impact of his parenting nor his perceived level of parental overprotection in this cohort. Moreover, we used this parenting measure and the relationship quality measure that were acquired at 24 months because we did not collect that data at the 6- to 8-year follow-up to reduce participant burden. This may have reduced our ability to detect the self-reported paternal association with perceived parenting as a potential mediator and relationship quality as a moderator of paternal mental health issues on child outcomes.

It is not clear what factors may account for the detected associations between paternal mental health and child outcomes. Our results did not support the hypothesis that maternal mental health, paternal self-perceived parenting toward the child at 24 months of age, or the couple's satisfaction measured when the child was 24 months of age would explain some of the variance in those associations. Further studies should consider how the trajectories of these potential factors, particularly those measured in temporal proximity to the childhood assessments, might better explain the relationships between paternal mental health and child cognitive and behavioral outcomes.

The associations between higher paternal mental health symptoms higher cognitive performance and fewer behavioral difficulties in the child were unexpected and present an apparent paradox considering the existing body of literature. We hypothesized that higher paternal mental health symptoms would be associated with poorer cognitive and behavioral outcomes, as previously documented (Ramchandani et al., 2005; Ramchandani and Psychogiou, 2009) (see Ashraf et al., 2023 for a recent review and Cui et al., 2020 for a recent meta-analysis). The associations reported are consistent with a small number of studies reporting positive relationships between paternal factors and child behavioral symptoms. For example, Mezulis et al. (2004) found that paternal depression, in the context of fathers who spend moderate-to-high amounts of time with their infants, can exacerbate the adverse long-term associations between maternal depression and child behavioral problems, and they also reported another interesting interaction where fathers can buffer adverse associations between maternal depression and child behavioral problems. They found that in the context of maternal depression, father's parenting styles (i.e., low-to-medium-high-warmth involvement and high amounts of medium-high control) were associated with lower internalizing behaviors in the child, suggesting that fathers might buffer the effect of high maternal depression on child behavioral problems. The sample size of our study precluded our ability to test complex interactions, but follow-up studies, even within the 3D-Transition cohort, may be able to address these interactions.

Strengths of this study include its ability to assess the relative developmental association of prenatal vs. childhood parental mental health symptoms on the child and the range of the standardized assessments, which cover several facets of parental mental health and children's cognitive and behavioral outcomes, from pregnancy to middle childhood. Few pregnancy cohorts can reach this level of detail when collecting both maternal and paternal data, and subsequent measures of children's cognition and behavior. Other strengths include the detailed measurement of sociodemographic, educational, substance use, parenting perceptions, and marital relationship quality in both parents, during pregnancy and in the perinatal period, allowing us to consider a long list of potential control variables. While one prior investigation of paternal developmental effects included the child's expressive vocabulary as an outcome measure (measured using the MacArthur Communicative Development Inventories) and found detrimental effects of paternal middle childhood levels of depression (Paulson et al., 2009), this study examined younger children (2 years old) and only measured expressive vocabulary. In contrast, the WISC-V is standardized and thus regarded as an accurate assessment of general intelligence, covering a wide range of cognitive functions including verbal comprehension, fluid reasoning, working memory, visual-spatial performance, and processing speed (Canivez et al., 2016). As such, we are contributing novel data supporting the importance of paternal mental health on cognition during middle childhood, as measured by a standardized cognitive battery.

Despite these strengths, it is important to note that our findings are not congruent with those of other studies that have investigated the role of the father in child development. These prior studies differed from ours in several ways, notably regarding sample characteristics and methodological aspects, with our sample showing an overall higher SES and less severe mental health symptoms in the parents. Similarly, children in our sample scored within normal ranges on all SDQ subscales (Shojaei et al., 2008). Since this questionnaire was completed by the parent, it is therefore possible that parents who rated higher mental health symptoms may have been more attuned to their child's symptoms. In addition, prior studies did not distinguish between paternal depression and anxiety or use cutoffs to characterize the presence vs. absence of depression/anxiety in parents based on self-reported screening questionnaires, whereas, in this study, mental health symptoms were studied as continuous variables. Of note, anxious/depressive symptoms in our sample were primarily non-clinical, which increases generalizability to non-clinical populations, but may have made it more difficult to detect associations between paternal mental health and child outcomes. While our sample size is small, the potential generalizability of our findings to those of other pregnancy cohorts is supported by the large and expected adverse effects of maternal anxiety and depression symptoms on child development. However, recruited participants did not differ significantly from those of the larger 3D and 3D-Transition samples in terms of parental age, education, or ethnicity. Furthermore, results remained significant even after accounting for maternal mental health issues and parental education, suggesting that, at the very least, the effects of paternal anxiety/depression symptoms on child cognition may be non-linear.

The difference between the measures used to assess prenatal and concurrent anxiety and depression assessments in middle childhood represents another limitation. However, pre- and concurrent mental health symptoms were highly correlated, and all scales have been validated for use in perinatal populations (Wilcox et al., 1998; Tandon et al., 2012). Social desirability bias has been shown to bias parents' reports of both their behavior toward their children and their relationship satisfaction (Bennett et al., 2006; Bornstein et al., 2015; Visschers et al., 2017). This may influence any self-report measure of parent–child parenting perception and marital relationship quality and decrease reliability and validity of such measures, such as naturalistic observations of the family interactions, may be needed to determine the effects of parent–child parenting perception and marital relationship quality. Finally, child behavior was rated on the SDQ by fathers or mothers. Even though no significant differences were found in SDQ scores of children rated by their father vs. their mother, we cannot entirely exclude that part of the variance contributing to findings related to child behavior may be related to sex- or gender-based factors in parental reports of their child's behavior.

Another limitation of the study is that although greater paternal depressive and anxiety symptoms were associated with higher child IQ, we did not measure parental intelligence/IQ as a potential confounder. Literature shows that parental IQ is associated with child IQ; however, this association is low in infancy, increases in childhood but remains weak, and then becomes much stronger in adolescence and adulthood (Whitley et al., 2011; von Stumm and Plomin, 2015). Although we did not take parental IQ into account, we did account for other factors that are related to child IQ, such as parental SES (von Stumm and Plomin, 2015) and parental education (Cave et al., 2022).

## 5. Conclusion

Children with fathers who self-reported higher anxious or depressive symptoms during pregnancy or middle childhood performed better on a standardized cognitive assessment battery and exhibited fewer behavioral difficulties at school entry, over and above any associations with maternal mental health and parental education. Continued research on the complex interactions between biopsychosocial factors related to maternal and paternal mental health, across a spectrum of symptom-severity (i.e., subclinical and clinical) on long-term child social, emotional, and cognitive development is needed.

## Data availability statement

The data are under the jurisdiction of the CHU Sainte-Justine Research Ethics Committee and subject to current provincial and national privacy laws guiding their ethical use in Québec. To submit a request, please visit http://www.irnpqeo.ca/en/researchers/ or contact the corresponding author.

## Ethics statement

The studies involving humans were approved by Research Ethics Board (REB) at the McGill University Health Centre (MUHC) and the CHU Sainte-Justine Mother and Child University Hospital Center. The studies were conducted in accordance with the local legislation and institutional requirements. Written informed consent for participation in this study was provided by the participants' legal guardians/next of kin.

## Author contributions

T-VN, JRS, and WDF conceived and designed the Paternal Study. T-VN, CC, and SLJ conceived this sub-analysis study. CC and JL performed Paternal Study data collection and data entry. GE managed the database with input from CC, JL, JRS, SLJ, and T-VN. CC performed statistical analyses in consultation with GE, SLJ, and T-VN. GE provided statistical consultation and performed *post-hoc* analyses. CC wrote the first draft of the manuscript as part of a Master's thesis in consultation with supervisor T-VN and SLJ. TCM, KPD, JL, IG, CHR, and KH assisted in preparing drafts of the manuscript for submission. TCM oversees all ongoing aspects of the Paternal Study database. SLJ and TCM led the revisions of the article through to submission and publication. All authors contributed to revisions, intellectual content, and approved the manuscript submission.
